# S7-3 The contribution of walking policy in Ireland to the global sustainable development agenda

**DOI:** 10.1093/eurpub/ckad133.035

**Published:** 2023-09-11

**Authors:** Dylan Power, Barry Lambe, Niamh Murphy

**Affiliations:** Centre for Health Behaviour Research, South East Technological University, Waterford, Ireland. Get Ireland Walking, Dublin, Ireland; Centre for Health Behaviour Research, South East Technological University, Waterford, Ireland; Centre for Health Behaviour Research, South East Technological University, Waterford, Ireland

## Abstract

**Purpose:**

Increasing population levels of walking holds benefits for public and planetary health. While individual level interventions to promote walking have been shown to be efficacious, upstream interventions such as policies harness the greatest potential for impact at the population level. However, little is known about the nature and presence of walking policy in Ireland and the extent to which it aligns to national and global goals. This research provides an overview of a policy analysis of walking policy in Ireland.

**Methods:**

This study used multiple methods to provide a critical overview of walking policy. Firstly, a six-phase process was employed to conduct a content analysis of local and national walking policy in Ireland. Secondly, conceptual linkage exercises were conducted to assess the contribution of walking policy in Ireland to Ireland’s National Strategic Outcomes and the United Nations Sustainable Development Goals.

**Results:**

Overall, half (n = 13) of the counties in the Republic of Ireland were found to have no local level walking policies. Walking was identified to hold the potential to contribute to over half (n = 7) of the United Nations Sustainable Development Goals. Ireland’s only national level walking specific policy, the Get Ireland Walking Strategy and Action Plan 2017-2020, was identified to potentially contribute to four of Ireland’s National Strategic Outcomes and three United Nations Sustainable Development Goals. There were no actions within national walking policy in Ireland which were identified as contributing to SDG 13 Climate Action.

**Conclusions:**

Multidisciplinary action is required to update walking-related policy with embedded evaluation and governance mechanisms in all local walking systems. Furthermore, analysing walking policy in Ireland through the lens of the Sustainable Development Goals has informed the direction of future walking policy in Ireland. As of January 2023, the results of this research have shaped the next iteration of Get Ireland Walking’s national policy to ensure alignment with global sustainable development goals.

**Support/Funding Source:**

This research was co-funded by South East Technological University and Get Ireland Walking.

